# Transcriptional programs: Modelling higher order structure in transcriptional control

**DOI:** 10.1186/1471-2105-10-218

**Published:** 2009-07-16

**Authors:** John E Reid, Sascha Ott, Lorenz Wernisch

**Affiliations:** 1 MRC Biostatistics Unit, Institute of Public Health, University Forvie Site, Robinson Way, Cambridge CB2 0SR, UK; 2 Systems Biology Centre, Coventry House, University of Warwick, Coventry CV4 7AL, UK

## Abstract

**Background:**

Transcriptional regulation is an important part of regulatory control in eukaryotes. Even if binding motifs for transcription factors are known, the task of finding binding sites by scanning sequences is plagued by false positives. One way to improve the detection of binding sites from motifs is by taking cooperativity of transcription factor binding into account. We propose a non-parametric probabilistic model, similar to a document topic model, for detecting *transcriptional programs*, groups of cooperative transcription factors and co-regulated genes. The analysis results in transcriptional programs which generalise both transcriptional modules and TF-target gene incidence matrices and provide a higher-level summary of these structures. The method is independent of prior specification of training sets of genes, for example, via gene expression data. The analysis is based on known binding motifs.

**Results:**

We applied our method to putative regulatory regions of 18,445 *Mus musculus *genes. We discovered just 68 transcriptional programs that effectively summarised the action of 149 transcription factors on these genes. Several of these programs were significantly enriched for known biological processes and signalling pathways. One transcriptional program has a significant overlap with a reference set of cell cycle specific transcription factors.

**Conclusion:**

Our method is able to pick out higher order structure from noisy sequence analyses. The transcriptional programs it identifies potentially represent common mechanisms of regulatory control across the genome. It simultaneously predicts which genes are co-regulated and which sets of transcription factors cooperate to achieve this co-regulation. The programs we discovered enable biologists to choose new genes and transcription factors to study in specific transcriptional regulatory systems.

## Background

Organisms ranging in complexity from bacteria to higher eukaryotes are able to react and adapt to environmental and cellular signals. These responses are often encoded as complex gene regulatory networks. In these networks the expression of a gene's products is regulated by the activity of other genes. Although regulation can occur at many levels, we focus on transcriptional regulation, one of the most important and pervasive methods of regulation in eukaryotes. Transcriptional regulation occurs when certain gene products, transcription factors (TFs), bind to the DNA at binding sites (TFBSs) and affect the transcription of the regulated gene by modulation of the RNA polymerase complex. TFBSs often appear in clusters or cis-regulatory modules (CRMs), presumably to enable interactions between TFs binding there.

### Combinatorics of transcriptional regulation

TFs do not work in isolation from each other. Particularly in higher organisms, combinatorial operations are often necessary for the response of a cell to external stimuli or developmental programs. Such a response is frequently implemented as a transcriptional switch where a combination of presence or absence of certain TFs regulates the expression of a certain gene. Several well characterised examples of the coordination of TFs are known. For instance, a set of well studied TFs in *Drosophila melanogaster *that govern spatial patterns of development in its embryo is described in [1]; higher eukaryotes are known to use CRMs to integrate cellular signalling information [2]; the development of the anterior pituitary gland is regulated by combinatorial actions of specific activating and restricting factors [3] which determine cell type.

Conversely, cellular processes often involve the coordinated expression of sets of genes. Hence there is reason to suppose that not only do particular sets of transcription factors regulate particular genes but that these sets are also reused across the genome: that is, co-regulated genes are often targets of the same TFs. Genomic data commonly available today, such as sequence data, expression data or TF localisation data, do not permit direct inference of the higher order structure in transcriptional regulation. Most analyses of these data operate at the individual TF level. When the data permit it and the biologist is interested in this level of detail, it is certainly appropriate. However, genomic data is often noisy or incomplete. In this case a summary or view of higher order structure in transcriptional regulation is easier to interpret.

### Identification of binding sites by sequence analysis

The databases TRANSFAC [4] and JASPAR [5] hold the most widely used collections of position specific scoring matrices (PSSMs). Each PSSM is a probabilistic model of the DNA binding specificities of a particular TF: given the PSSM and a stretch of DNA the likelihood of that TF binding to different positions in the sequence can be computationally predicted. There are several problems with this approach: algorithms that find putative binding sites are known to generate many false positives; the regions in which regulatory TFBSs are located are not normally known in advance; and, unfortunately, JASPAR and TRANSFAC do not contain PSSMs for all TFs of interest. We chose to use the PSSMs in TRANSFAC for our analysis.

### Our model

Our model aims to discover cooperative effects between transcription factors in noisy sequence analysis data. We use a model that has had success in the field of document modelling where the task is to infer the latent topics that best summarise a corpus of documents. Each document is modelled as a mixture of several topics drawn from a shared pool of unknown topics and each topic is modelled as a collection of words. Only the documents are given as input to the model.

**Figure 1:**
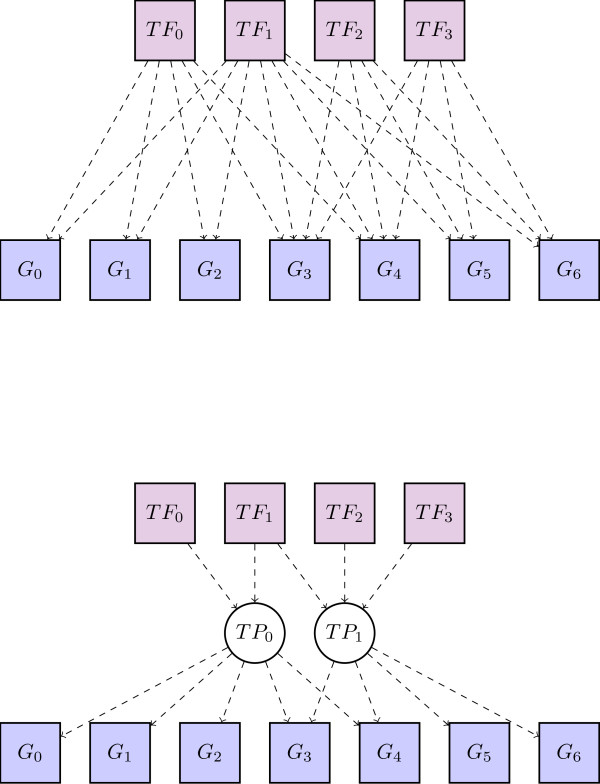
**Transcriptional programs**. Two schematics of the same regulatory network. Both representations have 4 transcription factors at the top and 7 genes at the bottom. The lower network uses latent transcriptional programs as intermediaries to reduce its complexity. Note that the transcriptional programs can overlap, for example *TF*_1 _is in both programs and that the same gene can be targeted by multiple programs, for example *G*_3 _and *G*_4_.

To explain the use of this model in the context of transcriptional regulation we draw an analogy: in our model a document is analogous to a gene; a word is analogous to a transcription factor and the occurrence of a word in a document is analogous to a binding site in a gene's CRM. To complete the picture, a topic is analogous to what we term a *transcriptional program *(TP). A TP captures the notion of a set of transcription factors that act in a coordinated manner across a set of target genes. So in the same way that a document's topics define its context, a gene's transcriptional programs summarise its transcriptional regulation. Figure 1 shows how transcriptional programs can summarise regulatory relationships.

Hierarchical Dirichlet processes (HDPs) are a natural framework to use in document-topic modelling and hence for our work in transcriptional regulation. In our framework, transcriptional programs are modelled as distributions over transcription factors. Each gene's transcriptional regulation is modelled as a mixture of these programs. Dirichlet process mixtures (DPMs) are a non-parametric technique for modelling mixtures where the number of components is unknown. We use DPMs to model the mixture associated with each gene's transcriptional regulation. In order to share transcriptional programs between genes we use a common base distribution for the DPMs which is itself a DPM. This step makes our model hierarchical. An extensive review of HDPs is given in [6].

### Previous work

Quite a few approaches have been suggested in the literature to identify groups of TFs that co-regulate genes, often called transcriptional modules (TM). They all differ from our approach in several respects. One major difference is that our concept of transcriptional programs (TP) is slightly more abstract than TMs. A TM is often defined as a set of TFs that physically bind next to each other in the vicinity of the regulated gene. Many approaches either enumerate all possible combinations of TFs up to a certain number (less than half a dozen or so) as potential TMs and search for over-representation of TMs in various groups of genes [7-10]. There is usually a computationally intensive post-processing step involved in clustering TMs according to *ad hoc *rules in these combinatorial approaches to reduce the number of highly similar TMs. Alternatively, an incidence matrix (or bipartite graph) is calculated linking each TF to the genes it regulates [11-13] (see Figure 1 top).

In contrast, a transcriptional program, as we define it, comprises TFs as well as genes (see Figure 1 bottom) and does not necessarily require a physical vicinity of binding sites for all the TFs in the program. For example, if two transcriptional modules have some common TFs, not necessarily sharing all of them, they might be merged into one transcriptional program by our algorithm. Whether this happens depends on the amount of overlap and the number of co-occurrences of their TFs. In a way, transcriptional programs generalise both transcriptional modules and TF-gene incidence matrices and provide a higher-level summary contained in these structures. To our knowledge, the only other work defining transcriptional programs in a similar way is by Tanay et al. [14]. In contrast to their work, where such programs are found by enumeration, scoring and filtering, we model transcriptional programs explicitly within a comprehensive probabilistic model.

Some work, as discussed below, insists on clusters of co-regulated genes or groups of co-regulating TFs to be disjoint. Our approach is open to the possibility that genes as well as TFs can be members of several TPs simultaneously. A further difference is that many approaches require a positive gene set, for example, by co-expression, as well as a background, set to detect TMs that characterise one set against the other. Our approach is essentially an unsupervised one, where TPs are discovered from one sequence set. This is a more challenging problem but it requires less input from the user and avoids problems of mis-identification of the positive set.

To our knowledge, our approach is the first application of a document topic model to transcriptional regulation. Such models have the distinct advantage of using very few free parameters that need to be specified.

Being more specific about previous work, CREME [7] uses a sliding window to look for combinations of transcription factor binding sites that are over-represented in promoters of the genes of interest. Only combinations whose sites are physically close to one another can be detected in this way. The user must specify the maximum number of factors in a promoter. oPOSSUM2 [8] looks for pairs and triplets of transcription factors that are over-represented in the promoters of the genes. TREMOR [10] is similar but uses the Mahalanobis distance to distinguish between similar PSSMs that represent different members of the same family of transcription factors. It also removes some dependence on arbitrary *p*-value thresholds.

All of these methods discriminate between a positive user-specified set of genes and a negative (background) set. Our method differs in that it fits a model of the entire set of genes at once.

Kreiman [9] looks for over-representation of combinations of up to 4 TFs in co-expressed genes. Bluethgen et al. [15] use Cluster-Buster [16] to identify groups of potentially co-regulating TFs which are then further filtered by statistical enrichment of classes of regulated genes in the Gene Ontology (GO) catalogue [17]. There is some work that integrates more than one data source. Some combination of ChIP-chip, binding site analysis (either *de novo *or PSSM-based) and expression data are commonly used. Heuristics or probabilistic models are used to search for consistent structure amongst these data sources. Almost all this work has been carried out in *Saccharomyces cerevisiae*. ReMoDiscovery [11] builds on the Apriori framework in a two-step procedure which examines expression profiles and ChIP-chip data. MOFA [18] combines binding data with time-series microarray data to build transcriptional modules and explicitly models which TFs up or down-regulate which genes. SAMBA [14] is a biclustering framework that analyses gene expression, protein interaction, growth phenotype, and TF binding data. In COGRIM, Chen et al. [12] use Gibbs sampling in a Bayesian hierarchical model to integrate expression data, PSSM analyses and ChIP-chip data. They model uncertainty in each data source independently but each module is associated with exactly one transcription factor. As discussed above most of this work is reconstructing pair relationships of TFs and regulated genes.

Segal et al. [19] have integrated a motif search algorithm and gene expression data to find motif profiles (analogous to transcriptional programs) in *Saccharomyces cerevisiae*. Their model partitions the genes into a fixed number of mutually exclusive sets which have the same expression pattern across experiments. Each gene is the target of exactly one motif profile, hence their model does not allow so much structure in the latent profiles/programs. Also, the number of partitions must be fixed somewhat arbitrarily in advance by the user. They focus on *Saccharomyces cerevisiae *which has a simpler transcriptional code than *Mus musculus*, the focus of our study.

Various other probabilistic models that require specification of the number of modules by the user have been implemented. Xu et al. [20] build on the module networks of Segal et al. [21]. These models also partition the gene set to find transcriptional modules. Our model allows genes to be the target of more than one transcriptional program.

Other algorithms also use non-parametric probabilistic models to obviate the need to specify the number of modules. Gerber et al. [22] use hierarchical Dirichlet processes to discover expression programs in human microarrays. They use a similar model to ours, except their data are discretised expression levels rather than putative TFBSs. They use a Markov chain Monte Carlo (MCMC) method for inference which takes an order of magnitude longer than our variational approach. The MCMC method produces a posterior distribution over the unknowns in their model. One of the latent variables in their model is the structure of the gene hierarchy. Identifiability issues force them to use a complex set of heuristics to summarise this hierarchy. Liu et al. [23] use a Bayesian hierarchical model to examine yeast gene expression and ChIP-chip data. Their extension of an infinite mixture model limits each program to represent binding data for at most one transcription factor. It is difficult to see how cooperative effects are estimated by the model.

## Results and discussion

We analysed the promoter regions of 18,445 *Mus musculus *genes using PSSMs from TRANSFAC. This generated 78,085 putative TFBSs of 149 TFs which scored above a stringent threshold (see Methods). We ran our model on these putative TFBSs and it discovered 68 latent transcriptional programs.

**Figure 2:**
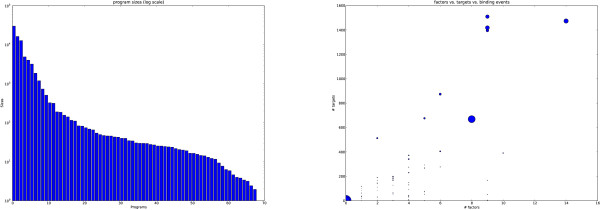
**Program sizes**. We show how many TFBSs are generated by each transcriptional program in our model and relate this to the number of target genes and TFs associated with each program. On the left, the number of binding sites that our model predicts are explained by each program is shown on a log-scale. A by-product of our algorithm is that the programs are sorted by the number of TFBSs they are responsible for. The most frequently used transcriptional program accounted for almost 30,000 and 15.000 binding sites respectively and the smallest just for a handful. The largest programs are composed predominantly of common transcription factors and in general the smaller programs explain occurrences of rarer transcription factors. The right is a scatter plot of the programs showing the number of TFs against target genes. The area of each scatter point is proportional to the number of binding sites it is responsible for. Note the first two programs do not have any genes or targets associated with them, their distributions over TFs are very similar to the genomic distribution and they are ubiquitous.

The number of TFBSs explained by each of the 68 programs varied considerably. Most of the TFBSs were explained by the largest 10 programs (Figure 2). As demonstrated in the GO enrichment validation (Table 1) our model was able to find significant signals in those programs that accounted for many TFBSs as well as those that accounted for few.

**Table 1:** Interesting transcriptional programs.

TP	# Targets	Factors
2	669	Nkx2-1 Dbp Ahr Srebf1 Egr2 Tcfap2a Sp1 Egr1
3	1474	Rest Pparg Pax6 Creb1 Vdr Ets1 Hivep1 Pbx1 Dmrt2 Hand1 Dmrta1 Irf8 Atf2 Ar
4	1419	Gabpa Gzf1 Ppara Stat3 Hoxa5 Ikzf1 Hnf4a Srf Pax5
5	1510	Atf4 Dmrt1 Lhx3 Nkx6-1 Stat5a Runx2 Irf2 Pax4 Pax1
12	198	Nfya E2f1 Mtf1
13	372	Foxo3 Foxj2 Dmrt3 Nr2f1
15	230	Pbx1 Nr5a1 Sry Rora
18	268	Pou1f1 Pax2 Ets2 Cux1 Tbp
25	275	Srf T CT025657.12-201 Pou5f1
28	167	Cebpa Gabpa Cebpg Dbp Tgif1 Atf3 Rela Hes1 CT025657.12-201
53	111	Gzf1 Atf2
55	54	Klf4 Prdm1 Atf3

We present the factors of the transcriptional programs highlighted in the validation section of the results.

**Figure 3:**
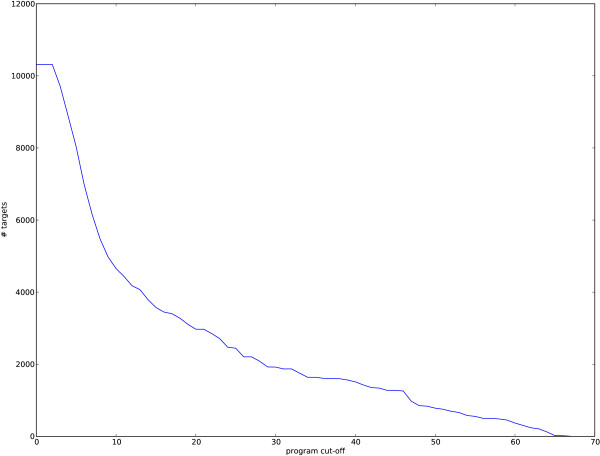
**Number of target genes of programs by size**. We plot how many genes are targeted by the programs smaller than a given size. The programs that account for more binding sites are less interesting in terms of cooperative effects, so we plot the size of the set of all targets of all programs smaller than a given size. The size cut-off varies along the *x*-axis (indexed by program) and the *y*-axis represents the total number of genes targeted by those programs. For example, excluding the first 10 programs, just over 4,000 distinct genes are targeted by the remainder of the programs.

As the model associates each TFBS with a program, even those TFBSs for which co-operative effects cannot be found must be associated with a program. The model uses the largest two programs (programs 0 and 1) for these TFBSs: their distribution over factors is vague and they target many genes. To some extent, the programs that explain more binding sites are less likely to represent true cooperative effects. We looked at the number of target genes of the programs in this context. That is, we analysed the total number of target genes of all programs smaller than a given size (Figure 3). Including the first two vague programs, a total of over 10,000 genes are associated with our programs. Most of the binding sites are explained by the first 10 programs and using this as a cut-off we can see that the remainder of the programs still target over 4,000 genes. This is a sizable proportion of the genome that can be strongly associated with the cooperative combinations of factors defined by our programs.

**Figure 4:**
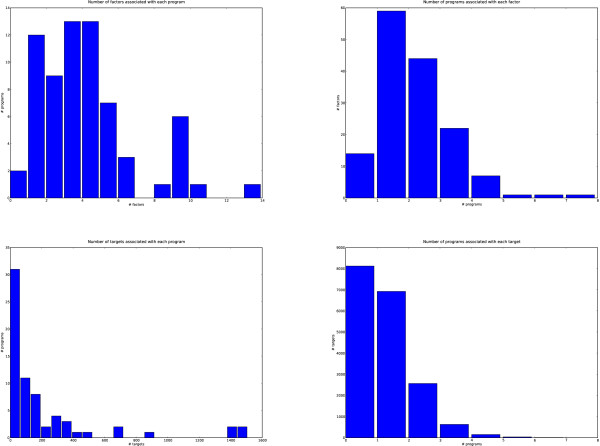
**DPM summaries**. Each transcriptional program is associated with a set of TFs and a set of target genes. We examined the relationships between the programs and their targets and factors. The top left figure shows that most programs have fewer than 7 factors associated with them. The top right illustrates that most factors are in fewer than 5 programs. The bottom left shows that a few programs target many genes but most programs have fewer than 200 targets and the bottom right demonstrates most genes are targeted by 2 or fewer programs.

**Figure 5:**
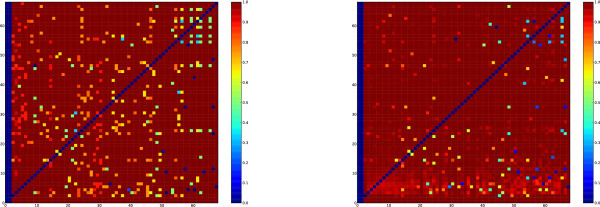
**Program intersections**. The intersection of all the programs' factors and targets. On the left, we show how much the factors of the programs overlap. This is represented as the ratio of the size of the intersection between the factors to the size of one of the sets of factors. The right is the same analysis of the programs' targets. The overlap between targets and factors is negligible in almost all cases. The sets of factors that do overlap to some extent are those that are not responsible for many TFBSs. The first two programs do not have any factors or targets associated with them.

In general, we found a good separation between the programs, in that any given TF or gene is unlikely to be associated with many programs and conversely that most programs were associated with a small number of TFs and genes (Figure 4). This was confirmed by our analysis of the intersection between pairs of programs' TFs and the overlap between their target genes (Figure 5).

### Validation

In order to test whether the transcriptional programs capture real biological structure we validated the TPs using an analysis of enrichment for GO terms [17], signalling pathways from the KEGG database [24], tissue specific co-expressed genes from SymAtlas [25], and groups of known interacting TFs from the literature. We present those transcriptional programs that were noteworthy in the validation in Table 1. All of the factors and targets associated with all the programs are presented as Additional File 1.

#### GO term enrichment

Each program is associated with a set of transcription factors and a set of target genes. We tested the genes and the factors in each program for enrichment of terms in the biological process GO ontology. We used a standard hypergeometric test in conjunction with the weight method implemented in the top GO R-package [26] as a significance test. Table 2 shows the result of the GO enrichment analysis.

**Table 2:** GO enrichment.

TP	Factors	GO term		GO description	annotated	*p*-value
28	9	GO:0001889	BP	liver development	5/7	4.7e-06
						
TP	Targets	GO term		GO description	annotated	*p*-value

2	669	GO:0004842	MF	ubiquitin-protein ligase activity	16/121	8.7e-06
2	669	GO:0051216	BP	cartilage development	12/71	9.0e-06
3	1474	GO:0005550	MF	pheromone binding	11/27	2.8e-06
4	1419	GO:0005132	MF	interferon-alpha/beta receptor binding	7/8	1.2e-07
5	1510	GO:0005132	MF	interferon-alpha/beta receptor binding	6/8	7.3e-06
12	198	GO:0000786	CC	nucleosome	14/136	4.2e-10
12	198	GO:0005634	CC	nucleus	84/4115	8.2e-08
12	198	GO:0003677	MF	DNA binding	54/1993	4.0e-07
12	198	GO:0003697	MF	single-stranded DNA binding	6/37	4.2e-06
12	198	GO:0006334	BP	nucleosome assembly	14/147	1.9e-09
12	198	GO:0006260	BP	DNA replication	16/151	2.4e-06
13	372	GO:0004984	MF	olfactory receptor activity	50/1001	5.4e-09
13	372	GO:0007186	BP	G-protein coupled receptor protein signa...	81/1968	1.4e-09
15	230	GO:0034097	BP	response to cytokine stimulus	5/15	1.0e-06
18	268	GO:0004984	MF	olfactory receptor activity	43/1001	6.0e-11
18	268	GO:0007166	BP	cell surface receptor linked signal tran...	74/2606	2.4e-08
18	268	GO:0007608	BP	sensory perception of smell	10/90	7.9e-07
25	275	GO:0004556	MF	alpha-amylase activity	4/5	2.1e-07
28	167	GO:0007186	BP	G-protein coupled receptor protein signa...	38/1968	4.1e-06
55	54	GO:0032183	MF	SUMO binding	2/2	1.0e-05

We tested each of the factors and target genes associated with each of the 68 transcriptional programs for enrichment of GO terms across the three GO ontologies: biological process (BP), molecular function (MF) and cellular component (CC). The number of factors (resp. targets) associated with the program is displayed followed by information about the GO term. 'Annotated' shows the number of factors (resp. targets) annotated with the term in the program compared to the total numbers of factors (resp. targets) annotated with the term. The *p*-values are not corrected for multiple testing. Based on a bootstrap analysis described in the Methods section any *p*-value below 1e-04 might be deemed significant at the 0.01 level.

#### KEGG pathway enrichment

We tested the genes and the factors in each program for enrichment in signalling pathways defined in the KEGG database. After Bonferroni correction for multiple testing, we found no significant results. However, we did find a significant result in conjunction with our analysis of known interacting TFs from the literature.

#### SymAtlas enrichment

We tested the target genes in each program for enrichment in tissue-specific co-expressed genes from the SymAtlas dataset. Genes over-expressed in embryonic tissues were significantly enriched in the targets of transcriptional program 53. Program 53 accounts for fewer than 100 binding sites out of the 78,085 sites, yet was strongly predictive of membership of the group of over-expressed genes. This demonstrates the ability of our method to find small signals in large datasets.

#### Literature

We took well known sets of interacting transcription factors from the literature and looked for programs that contained them. We looked for sets of TFs associated with the liver, muscle development, and the cell cycle. The three factors in transcriptional program 12 (E2F, NFY, MTF1) contain two of the three transcription factors in our analysis that are known to regulate the cell cycle (E2F, CREB, NFY [27]).

When we tested the targets of program 12 for enrichment in the KEGG cell cycle pathway (without correcting for multiple testing) we obtained a p-value of 9e-4. The extra TF in program 12 that is not in our literature derived set, MTF1, has been implicated in the cell cycle [28] and as a co-regulator with E2F [29].

### Biological interpretation

Several of the discovered programs have well defined biological meanings. Not many of the factors of the transcriptional programs were significantly enriched for GO terms. However, program 28 did contain 5 of 7 TFs that are annotated with the term "liver development" in its nine factors.

Several of the target sets of the programs were strongly associated with different GO terms. In particular, program 12 was particularly enriched for genes with nuclear products and those that are involved in nucleosome assembly. Program 18 appears to be associated with the sense of smell as it has strong enrichment for "olfactory receptor activity" and "sensory perception of smell".

## Conclusion

Discovering structure in sequence analyses is a difficult task. We are limited by the set of PSSMs available, our inability to predict regulatory genomic regions and the high false positive rate of PSSM scanning. Out of the three sets of interacting TFs that are most cited in the literature, we only recovered one of them. However, our method is looking for structure in a much larger dataset than other methods and does not have a positive set and a negative set of genes with which to discriminate.

Our model does find significant structure in these analyses and it is reasonable to suppose that this structure underlies some mechanisms of transcriptional regulation. This is to be expected given our understanding of the underlying biology. A valuable property of our method is that it finds structure at both large and small scales.

We are working on expanding our model to include other data sources. We anticipate using ChIP-seq and ChIP-chip data when it is available for enough TFs, either in conjunction with sequence analyses or other data sources related to regulation such as expression data.

We have shown that non-parametric probabilistic models are useful tools for unsupervised learning in this context. Techniques for genomic data integration are just starting to be applied with success to higher eukaryotes and we believe HDPM models are useful non-parametric tools for this task.

## Methods

### Binding site analyses

We extracted 1,000 repeat-masked base pairs upstream of the mouse transcriptional start sites (assembly July 2007) as defined in the UCSC Genome browser [30]. After removing strongly repeat-masked sequences we were left with 18,445 sequences for analysis.

We extracted a set of PSSMs from TRANSFAC version 11.4 for which we could map the factors they represent onto Ensembl gene identifiers [31]. From each promoter we need an estimate of the number of times there is a binding site for that PSSM in the CRM as input to our HDPM.

A PSSM of length *K *induces a distribution over *K*-mers that models binding sites for the transcription factor(s) it represents. Each position is modelled independently in this distribution. We can represent the PSSM as a matrix, *P*, where *P*_*k*, *b*_represents the probability of seeing base *b *at position *k *in the PSSM.

Given a *K*-mer, *W *= *w*_1_...*w*_*K*_, and using a simple uniform background model we can calculate the log odds ratio *L*(*W*) between the binding site model (the PSSM) and the background model, , where *V *is a prior on how likely we believe binding to be.

In this work we used a threshold of -1.3 and a *V *of -4.7 (all logarithms to base 10). Our experience working with biologists has shown us that this is a reasonable threshold to use. Our model does allow for noisy data and should accommodate false positives in the large vague transcriptional programs that do not model cooperative effects. We were constrained by our computational resources from lowering this threshold significantly.

Up to this point we have been dealing with each PSSM independently. Unfortunately they are not independent as, for instance, there are many factors for which TRANSFAC has more than one PSSM. Two PSSMs for the same factor are very likely to represent TFBSs at the same location in a promoter. We do not wish our model to learn this strong correlation instead of true transcriptional programs. We therefore reduce our set of TFBSs by taking the highest scoring set of non-overlapping TFBSs.

### Topic document model

In the field of information retrieval HDPs [6] are often used to model latent topics in documents. We apply them to TFBSs in promoters to infer latent transcriptional programs.

Our model is best described generatively, that is, we describe how to sample a suitable transcription factor from our model given a target gene. We follow the description in [32]. A gene *g *is linked to a distribution over transcriptional programs, which is represented by a (possibly infinite) vector *θ*_*g *_= (*θ*_*g*1_, *θ*_*g*2_,...,*θ*_*gk*_,...), where *θ*_*gk *_is the contribution of program *k *to gene *g*. All *θ*_*gk *_sum up to one for each *g*. A program *k *in turn is linked to a similar distribution over transcription factors, that is, program *k *is represented by a vector *ϕ*_*k *_= (*ϕ*_*k*1_...*ϕ*_*kJ*_), where *ϕ*_*kj *_is the contribution of transcription factor *j *to program *k *assuming there are *J *transcription factors in total. All *ϕ*_*kj *_sum up to one for each *k*. To sample a random transcription factor for binding site *i *upstream of gene *g*, we first sample a multinomial random variable variable *z*_*ig *_~ Mult(*θ*_*g*_) which indicates the transcriptional program the factor is drawn from. Next, we sample a second multinomial random variable  taking the selected transcriptional program *z*_*ig *_into account. Sample *x*_*ig *_specifies which transcription factor binds at binding site *i *upstream of gene *g*.

When calibrating the model using data, the task is to infer posterior distributions for parameters *θ*_*g *_and *ϕ*_*k*_. In order to do this, we place conjugate Dirichlet priors on the parameter vectors and use a variational approach to approximate their posterior distribution (for details see [32]). More specifically, we set *θ*_*g *_~ Dir(*απ*) and *ϕ*_*k *_~ Dir(*βτ*). *α *and *β *are scalar strength parameters that control the variances of the *θ*_*g *_and *ϕ*_*k *_respectively. *π *and *τ *are vectors and represent their respective means.

We do not wish to constrain our model to use a fixed number of transcriptional programs. Instead, we use a non-parametric approach where we allow a countably infinite number of transcriptional programs. Now *θ*_*g *_and *π *are infinite dimensional vectors. *π*_*g *_is modelled using an explicit stick-breaking construction [33] where *γ *controls how many transcriptional programs are used. Formally, the stick-breaking model is defined by



**Figure 6:**
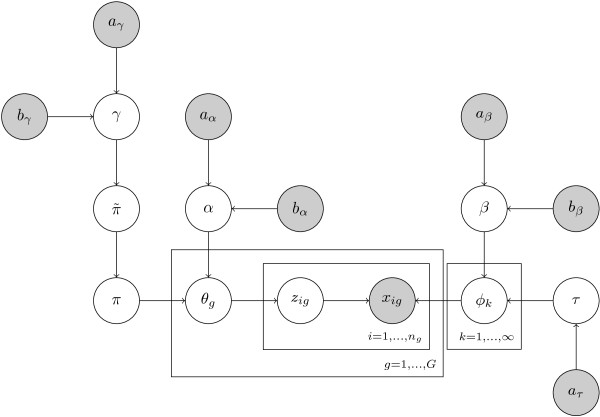
**HDPM model**. We present our model graphically. The shaded nodes represent observed variables (or equivalently from the model's perspective, fixed hyper-parameters). The clear nodes are the latent variables in the model. The boxes are called *plates*. If a node is inside a plate, its corresponding variable has a multiplicity equal to the size of the plate. For example there are *G *instances of the *θ*_*g *_variable as its node is inside the *g *= 1,...,*G *plate. See the text for a description of the variables.

Intuitively, probabilities *π*_*k *_are obtained by starting with a stick of length 1, and continuing to break pieces off it, their lengths representing the probabilities *π*_*k*_. Even if continued indefinitely the pieces all sum up to one, the total length of the stick, forming a proper probability distribution over the natural numbers. The size of piece *k *is determined as a fraction  of the remaining stick, whose length is , where  is a random number from the interval [0.1] distributed according to a Beta distribution. We also place priors on all the other hyperparameters of the model,



Our model is presented graphically in Figure 6.

### Inference

We implemented the collapsed variational inference technique described in [32] complete with the Gaussian approximation for non-zero counts.

### Thresholding the posterior

In our model each transcriptional program is represented as a distribution over factors, *ϕ*_*k*_, and each gene can be summarised as a distribution over programs, *θ*_*g*_. In order to examine the programs we have learnt, we thresholded these distributions to discover which programs are over-represented in which genes and which factors are over-represented in which programs. However, due to the collapsed nature of the inference algorithm we do not directly obtain a posterior over them as they have been integrated out. The inference algorithm does infer which factors have binding sites in which genes due to which transcriptional programs. These inferences are summarised as the expectations of various counts and these allow us to estimate the *θ*_*g *_and *ϕ*_*k *_and hence associate transcriptional programs with genes and with transcription factors.

More formally, in an analogous notation to [32], we define *n*_*gkf *_as the number of binding sites for factor *f *drawn from transcriptional program *k *in the promoter of gene *g*. A'.' in the subscript indicates summation over that index. For example *n*_.*kf *_is the number of binding sites of factor *f *drawn across all genes from program *k*. Now we make point estimates:



We define  to be the empirical distribution of factors. Now we associate with transcriptional program *k *all those factors, *f*, for which . Likewise we define  and associate those genes, *g*, with transcriptional programs, *k*, for which . We found our method was insensitive to the actual choice of threshold: when we varied it between 1.5 and 10 the results were not affected significantly.

### Validation

Correcting for multiple testing in a GO ontology is difficult due to its hierarchical nature. To validate the strength of our results we generated random samples from the same populations of genes and transcription factors to test for enrichment. We choose sample sizes to cover the range of sizes in the discovered transcriptional programs. For each size we sampled 100 independent sets and calculated the exponent of the best *p*-value found in an GO term enrichment analysis.

### Significance of *p*-values

**Figure 7:**
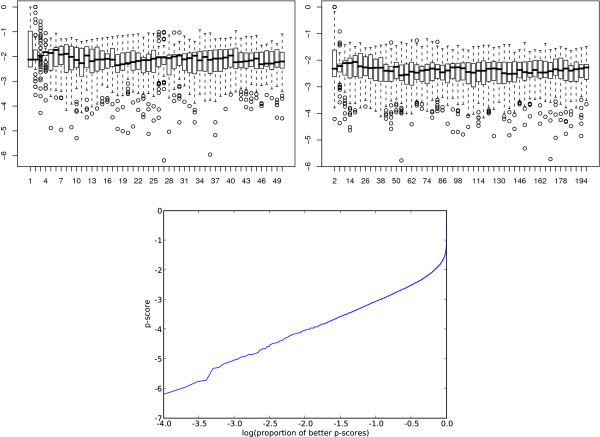
**Bootstrap**. Assessment of the significance of the results from the GO enrichment analysis by random samples of factors and genes. We show a boxplot of the *p*-scores for the randomly sampled factors on the top left and the targets on the top right. The *x*-axes are the sample sizes and the *y*-axes are the *p*-scores. We sampled 100 times at each of 50 different sample sizes for the factors and the targets. The lower plot shows the sorted *p*-scores plotted against the base 10 logarithm of the proportion that are equal or better.

To assess the significance of the results from the GO enrichment analysis, we generated random samples of factors and genes. Each sample was analysed for GO enrichment using the same procedure as for the transcriptional programs that our model predicts. For each sample we take the best uncorrected *p*-value and refer to its base 10 logarithm as the *p*-score. In Figure 7 we show box-plots of the *p*-scores for the randomly sampled factors and target genes. We sampled 100 times at each of 50 different sample sizes for the factors and the targets. The sizes were chosen to reflect the range of sizes of the actual transcriptional programs. Hence each diagram represents 50 * 100 = 5000 independent samples. The sample size does not appear to affect the extreme value distribution of the best *p*-scores' exponents. From 10,000 independent samples, the lowest *p*-score is around -6. Plotting the sorted *p*-scores against the base 10 logarithm of the proportion that are equal or better gave us a good linear fit. This fit has an intercept very close to -2 which together with the linear relationship suggest adding 2 to the *p*-score to obtain a multiple testing corrected *p*-value exponent. That is a *p*-score of -6 would be equivalent to a *p*-value of 10 ^-4^.

## Authors' contributions

JR designed the PSSM scanning algorithm. He implemented the code for PSSM scanning, the topic-document HDPM and the analysis. He analysed the results and wrote the main body of this paper. SO helped develop the PSSM scanning algorithm. LW participated in the design of the model, the statistical analysis of the programs and wrote parts of the paper. All authors read and approved the final manuscript.

## Supplementary Material

Additional file 1 **Transcriptional programs – programs.tar.gz**. A gzipped archive containing the transcriptional programs output by our method. Click here for file
