# Supplementary material for: Transcriptional programs: Modelling higher order structure in transcriptional control
Source: BMC Bioinformatics. 2009 Jul 16;10:218. doi: 10.1186/1471-2105-10-218 (PMC2725141; doi:10.1186/1471-2105-10-218)
Supplement: Additional file 1 — Transcriptional programs – programs.tar.gz. A gzipped archive containing the transcriptional programs output by our method. [file 1471-2105-10-218-S1.zip › readme.html]

BioMed Central TeX template files


# BioMed Central TeX template  - Version 0.4 (January 27th 2006)

1 BioMed Central LaTeX template distribution

|  |  |
| --- | --- |
| bmc\_article.tex | The BioMed Central  manuscript template. Edit a copy of this file. |
| bmc\_article.bib | A sample BibTeX bibliography file used to create a .bbl by BibTeX using the bmc\_article.bst file |
| bmc\_article.bst | The BibTeX bibliography style file for the BioMed Central reference format |
| bmc\_article.cls | Page layout for for manuscripts. This makes small changes to the standard article template |
| readme.html | This document. |

 

## 

## 2 Requirements

2.1

The BioMed Central TeX template
should work with TeX distributions on any platform  it has been tested with
TeXShop on Mac OS
X and MiKTeX for Windows.

2.2

In order to submit a manuscript
as a .tex file to BioMed Central, you *must*

- use the BioMed Central template
- format your references with BibTeX using
  the bmc\_article.bst style file
- not rely on any non-standard macros,
  classes or files

If your TeX manuscript does not
meet the criteria above it will need to be converted to DVI format prior to
submission.

## 2 Guidelines for creating your manuscript using TeX

2.1

Follow the guidelines in the BioMed Central instructions
for authors given at http://www.biomedcentral.com/info/authors/

2.2

Make
sure your manuscript is compiled with LaTeX2e by using *documentclass*{..
. } and not *documentstyle*{. . . } in the preamble at the top of your
*.tex* document.

A template manuscript is supplied entitled *bmc\_article.tex* which
sets up the preferred page layout based on the standard *article.cls*. Two different styles are supplied; Review Style, which produces single column double spaced text, and Publication Style which produces two-column text. They can be toggled by commenting or uncommenting the relevant lines in the TeX file.

2.3

Make sure that you only a single *.tex* document
for the entire manuscript, as you will need to upload it as a single file (together
with its associated formatted bibliography file) . Do not use the \input command to include other .tex files.

2.4

The BioMed Central template uses the *cite.sty*
citation style and *url.sty* for url references. It also uses *ifthen.sty* and *multicol.sty* You should also use these
during the creation of your manuscript.

See

http://www.ctan.org/tex-archive/macros/latex/contrib/cite/cite.sty,
  
http://www.ctan.org/tex-archive/macros/latex/contrib/misc/url.sty  
http://www.ctan.org/tex-archive/macros/latex/unpacked/ifthen.sty  
http://www.ctan.org/tex-archive/macros/latex209/contrib/geomsty/multicol.sty

for these style files if they are not in your local
archive.

*cite.sty* turns [1,2,3,4] into [1-4].

*url.sty* formats urls so they can be broken down
cleanly when overflowing the right text boundary.

3 BibTeX

References *must* be formatted with BibTeX using
the BioMed Central style file.

i.e. when using the template, do not alter the command:
\bibliographystyle{bmc\_article}

The bibliography datafile is referred to with \*bibliography*{datafile1,
. . . , . . . }

The template makes use of a sample bibliography called
bmc\_article.bib  - you should update the \*bibliography* tag to
refer to your own bibliography.

4 Notes on uploading your manuscript

4.1

Make sure you are submitting only one *.tex* document
Please note that figures, large tables and any other
reference material should be submitted as separate files, not embedded in the
manuscript.

4.2

A *.bbl* file is generated when you use BibTeX
to format your article's reference list. It contains formatted details of all
references used in the manuscript. After uploading a TeX file to BioMed Central,
you will then be prompted to upload the *.bbl* file which goes with it..

## 5 The TeX article layout

This is the sectioning for a BMC-series Research Article l
manuscript submission . . .

 Abstract

 Background

 Results

 Conclusions

 Background

 Methods

 Results and Discussion

 Conclusions

 Authors contributions

 Acknowledgements

 References

 Figures

 Tables

 Additional files

## 5 Further information

The latest version of the BioMed Central TeX
template distribution, and full instructions on its use, are available here:

http://www.biomedcentral.com/info/ifora/tex/
